# Ozone and its derivatives in veterinary medicine: A careful appraisal

**DOI:** 10.1016/j.vas.2021.100191

**Published:** 2021-07-28

**Authors:** Jéssica Rodrigues Orlandin, Luciana Cristina Machado, Carlos Eduardo Ambrósio, Valter Travagli

**Affiliations:** a Department of Veterinary Medicine, Faculty of Animal Science and Food Engineering (FZEA-USP), University of São Paulo, Pirassununga, São Paulo, Brazil; b Department of Biotechnology, Chemistry and Pharmacy – Department of National Excellence 2018-2022, University of Siena, Italy

**Keywords:** Animal, Veterinary clinic, Ozone therapy, Ozone derivatives, Quality issues

## Abstract

The therapeutic use of ozone and its derivatives in the veterinary medicine it is still in an emergent stage. Gaseous ozone chemical instability makes necessary its extemporaneous preparation and the accordance about ozone treatments with the highest quality standards in publications is of paramount importance. Moreover, the numerous method of administration in different animal species, the prevalence of case reports, the deficiency of consistent evaluation of the outcomes, as well as the lack of standardization of the treatment operating procedures represents an open question for its spreading and official approval. The keywords “ozone”, “ozonated”, “ozonation” “ozonized”, “ozonization”, “oxygen-ozone therapy”, “veterinary”, “pets”, “animal” were used to perform a literature review using PubMed, Cochrane, Google Scholar, Zotero databases with the temporal restriction for published manuscripts starting from 2010. All the researches were critically evaluated, regardless of the impact factor, if any, of the journals in which they were presented. The deepening of the mechanisms of action of this bio-oxidative therapy can open new horizons on its use. The distinctive condition to achieve such a scenario is an improved knowledge of the qualitative/quantitative characteristics of ozone and its derivatives. All with the aim of taking nothing away to the cited original research papers, but of improving the promising therapeutic implications of ozone therapy in veterinary medicine as a standardization stimulus about this therapeutic resource with multiple application specificities.

## Highlights

• In the Vet field, there are lot of scientific publications with missing or incomplete data, poor case reports and papers without a control group or unrepresentative sample.
• Differences between animal and human blood composition, anatomy and physiology must be taken into consideration, especially in therapies not yet fully approved.
• Some practitioners produce their own ozonated solution or autohemotherapy, in the absence of information regarding the compatibility of the material used.
• Other than the properties of the commercial preparations, the standardization of both treatment methods and times influence the results obtained with ozone therapy.

## Graphical abstract

**Figure:**
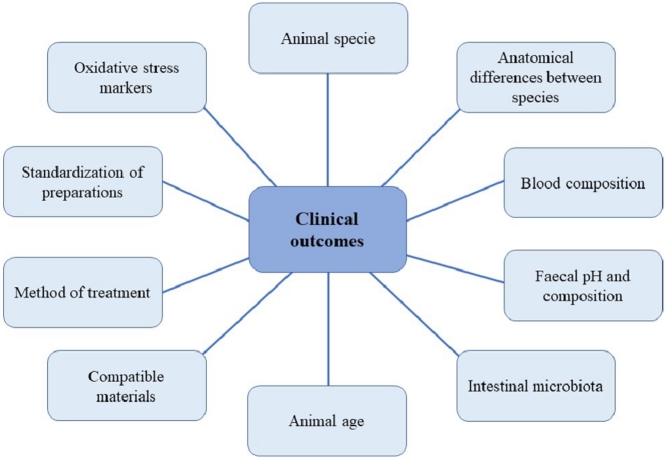

## 1 Introduction

The positive effects of ozone and its derivatives in health care is receiving more and more acclaim (Tricarico et al., 2020; Zanardi et al., 2016). However, there is divergence in the medical community on its use and benefits. The legislative part is also very varied, even banning the practice in some countries (“DOU 7/05/2020 - Pg. 269 - Seção 1 | Diário Oficial da União | Diários Jusbrasil,” n.d.; Mariño and Tapia, 2012; Thatiane et al., 2020).

Such aspects are very important because first it is central to differentiate ozone disinfectant power on surfaces and in environments with respect to its therapeutic activity (United States Enironmental Protection Agency, n.d.). On the other hand, oxygen-ozone therapy has several methods of application in veterinary practice (Bhatt et al., 2016; Duričić et al., 2015; Hayashi, 2018; Kozat and Okman, 2019; Nascente et al., 2019; Penido et al., 2010; Repciuc et al., 2016; Salazar Díaz, 2016; Samardžija et al., 2017; Sciorsci et al., 2020; Yiğitarslan et al., 2018). Although the results obtained are very promising and they confirm the potentiality of this therapeutic practice in the treatment of Vet diseases, the accuracy of the information of ozone application modalities becomes of vital importance in consideration of the refutations found in the approval of ozone therapy. In fact, ozone cannot be assimilated to any other drug, although the biochemical basis for its Veterinary use has been recently reviewed (Sciorsci et al., 2020; Tsuzuki et al., 2015). This statement is mainly justified by the following reasons:

i) Ozone chemical instability makes necessary its extemporaneous preparation. This inhibits obtaining ozone marketing authorization as a guarantee of the quality of medicinal gases, despite it represents not more than 5% of the gaseous mixture, while at least 95% is oxygen of medicinal grade (Travagli, 2020);
ii) The absence of ozone direct interactions at the receptor level in the organism. Such a fact does not allow classification of ozone among the gasotransmitters (Althaus and Clauss, 2013);
iii) The lack of a univocal classification of ozone among prodrugs. Such an issue is due to ozone capability to directly react with blood components like phospholipids, lipoproteins, generating both hydrophilic reactive oxygen species (ROS, mainly hydrogen peroxide), and lipid oxidation products (LOPs, mainly alkenals) as downstream effector molecules (Smith et al., 2017). In this context, ozonated derivatives starting from unsaturated matrices represent a separate category. In fact, in this case the products obtained are relatively stable and their biological activity is related to the peroxidic chemical species with a 1,2,4-trioxolanic structure (Almeida et al., 2013).

Therefore, an analytical appraisal of the published results is an important step to avoid the assimilation of biased evidence. The present study will also address the operative protocols (in terms of ozone generators, ozone concentrations, ozone derivatives and so on) adopted by practicing veterinarians, confronting good and negative results with respect to ozone treatment specifications. The keywords “ozone”, “ozonated”, “ozonation” “ozonized”, “ozonization”, “oxygen-ozone therapy”, “veterinary”, “pets”, were used to perform a literature review using PubMed, Cochrane, Google Scholar, Zotero databases with the temporal restriction for published manuscripts starting from 2010. All the researches were critically evaluated, regardless of the impact factor, if any, of the journals in which they were presented. For homogeneity's sake, the terms “ozonation” and “ozonated” were used in this article. Although the World Federation of Ozone Therapy recently suggested to rename the technique of autohemotherapy as systemic indirect endovenous ozonotherapy (SIEVO) (Baeza-Noci et al., 2018), the former it is still very popular and it will therefore be used in this article.

The aim of this paper is to take nothing away, but of improving the original studies, which highlights ozone as a generator of beneficial effector molecules in veterinary medicine. In fact, only a standardization capable of avoiding variability in the methodology can limit different and opposing findings in the literature.

## 2 Presentation of the original articles analyzed

In this section, summaries of both usual Materials and Methods as well as Results are reported, in accordance with the topics to which the various published articles belong. In order to make reading easier, the specific part relating to the characteristics of use of ozone, where present, is specifically indicated at the end of each paper summary. In the event of discrepancies in the indication of the units of measurement and decimals, they will be uniformly expressed throughout the review.

### 2.1 Safety issues

Rodríguez et al. (2019) used 3 rabbits for testing dermal irritability and other 3 for testing ophthalmologic irritability of an official preparation as ozonated oil cream. For the dermal group, after the trichotomy, a 0.5 g of the cream was applied in the skin once and covered with an adhesive. After one hour, the skin was washed with 0.9% NaCl and signs of erythema and edema were observed. The skin was evaluated at 1, 24, 48 and 72 hours. Initially, a mild irritation was observed. However, it has been resolved after some hours. For the ophthalmic group, 0.1 g of the same cream was applied in the conjunctival sac and the eye was closed for 15 seconds. After one hour, the eye was washed with saline solution and evaluated at 1, 24, 48 and 72 hours, comparing with the non-treated eye and observing changes in the conjunctiva, iris and cornea. No irritation was observed in the animals. The only indication relating to the ozonated oil used is the percentage in the cream formulation, equal to 30%.

Jaramillo et al. (2020) divided 16 healthy horses into two groups: a control group, where the horses received only oxygen; and the treated group, where the horses received 1L of gaseous intrarectal ozone three times a week. All the treatments manual rectal emptying. The blood samples were taken weekly and one week after the last treatment. Clinically, besides the increased defecation frequency in the group treated with ozone, no difference was observed between both groups. Also, no significative differences were observed in biochemical evaluation, fibrinogen concentrations or ROS production. In the horses treated with ozone, a significative increase of red blood cells counts, hemoglobin concentration and packet cells volume were observed when compared with the baseline and control group, concluding that it is a safe application and improves oxygenation and metabolism of tissues. Ozone concentration of 10 μg/mL for two applications; 15 μg/mL for the next two and 20 μg/mL for the final six applications were used.

Chica (2020) in his thesis reports the collection of 400 mL of blood from the jugular vein of 14 clinically healthy horses (kit SANO3®) for performing autohemotherapy. After that, the blood was reinfused in the vein. The animals were evaluated 24 hours before the first application, 24 hours after each five applications and each 15 days for 7 months. The interval between the applications was not informed. No side effects were observed in the horses and all the biochemical parameters observed were within physiological limits. For the first and second groups, the blood was ozonated with 250 mL of ozone at a concentration of 60 µg/mL and 25 µg/mL, respectively. The control group did not have the blood ozonated.

### 2.2 Antimicrobial evaluation

Madan et al. (2010) divided 27 rabbits affected by Dermatomycosis into six groups, treated with: placebo; industrially prepared topical ozonated oil; lab-scale ozonated citronellal at the concentration of 1%, 3%, and 5% in hydroalcoholic formulations; pure citronellal, at 5% in the same formulation. All the treatments lasted 15 days. The treatments based in 1% and 5% ozonated citronellal and placebo did not prove to be effective against Dermatomycosis. On the other hand, the animals treated with ozonated oil or 3% ozonated citronellal were completely healed after 20 days of the beginning of the protocol. Peroxide indexes of 596 and 2015 mmol equiv O_2_/Kg for the ozonated raw materials have been reported.

Daud et al. (2011) infected 18 rabbits with *Microsporum canis* in four different regions of the body. After seven days, one region was treated with 0.12 g of terbinaphine 1% cream; other two regions were treated with 0.12 g of ozonated oil; and the last region was not treated. The topical applications were performed once a day for 28 days. Besides terbinaphine cream was more effective in the fungus elimination, ozonated oil was also able to improve the lesions and fungicide effect against this dermatophyte. No further information was reported for the ozonated oil, apart from its trade name and provenience.

Roman (2015) in a paper published in a print magazine about alternative medicine as a forum for the entire alternative medicine community, administrated subcutaneous ozonated saline solution and performed rectal insufflation in 8 dogs and cats. After that, fecal sample from yard or litter box was collected immediately after defecation from 8 dogs and cats. After that, a fecal transplant was performed, orally and rectally. After the treatment, the patients showed improvement of the clinical signs. No further information was reported for the ozonated saline solution. The supposition that microbiome restorative therapy along with ozone therapy could be beneficial in treating medical conditions appears difficult to appreciate.

### 2.3 Theriogenology and reproductive medicine

Maldonado et al. (2017) selected 84 cows diagnosed with subclinical endometritis. 50 cows were treated with 60 mL of intrauterine ozonated distilled water, while the others 34 were treated with 500 mg intrauterine benzathine cephapirin. The group treated with ozone showed reduction in the percentual of polymorphonuclears cells and a better conception rate, when compared with the control group. The Authors state ozone concentration of 45 μg/mL in 60 mL of sterile distilled water.

Djurucic et al. (2012) performed an experiment using 96 cows diagnosed with retained fetal membranes, were 24-36 hours after parturition, they received once: intrauterine foam spray ozone for 5 seconds; six intrauterine ozone pearls. Other 47 cows without retained fetal membranes were used as control group. Insemination protocols were performed in all animals after 45 days. The cows treated with ozone had a similar or improved reproductive performance when compared with the control group. Commercially available foam and pearls containing ozone derivatives have been applied.

Similar results were observed by Đuričić et al. (2014), that divided 91 cows into three groups: metritis diagnosed on day 5 and/or 15 after calving; endometritis diagnosed on days 25 and/or 45 after calving; and animals with no signs of uterine inflammation. In all animas diagnosed with metritis, a single intrauterine foam spray was performed for 5 seconds. Animals with metritis treated with ozone had a shorter interval of days open until pregnancy and days until the first insemination following calving. Commercially available ozonated foam was applied.

Zobel et al. (2012) selected 1219 cows diagnosed with urovagina and treated them with: 100 mL of sterile 0.9% NaCl solution; 5g/100 mL of streptomycin; 10 mL of ozone spray intravaginal and 10 mL intrauterine. Artificial insemination was performed 10 minutes after the treatment. Animals treated with ozone had a shorter interval of days open, the fewest number of inseminations until pregnancy and the smallest number of culled cows, concluding it was the most effective treatment for urovagina in dairy cows. A commercially available pressurized ozone product has been applied.

For this study, Polat et al. (2015) used 53 cows with no clinical signs of metritis that were not pregnant, even after at least two artificial insemination. The animals were treated with intrauterine ozone foam spray or intrauterine rifaximin foam spray. After the first natural oestrus, the cows were artificially inseminated. Valuating the number of open days and number of artificial inseminations until pregnancy, ozone proved to be as effective as rifaximin on fertility in cows. Commercially available intrauterine ozone foam has been used.

Djuricic et al. (2015) treated 41 dairy goats diagnosed with retention of fetal membrane with intrauterine ozone foam spray for 2-3 seconds, or intrauterine oxytetracycline tablets. No statistical difference was observed between those two groups, and in both cases, animals were able to mate and gestate in the next spring season. Commercially available intrauterine ozone foam has been used.

Basically, the same study was performed by Đuričić et al. (2016) but this time in 256 sheep: 139 with dystocia and 49 with retained placenta. Animals were treated or with marketed intrauterine ozone foam spray for 2-3 seconds, or intrauterine tablets of oxytetracycline hydrochloride. Other 70 sheep with physiological puerperium were used as control group. Animals treated with ozone had similar reproductive performance when compared with the control group and better results when compared with animals treated with antibiotics.

Escandón et al. (2020) allocated 80 clinically healthy cows into two groups: treated with 50 mL intrauterine ozonized distilled sterile solution 35 days after calving; and non-treated cows as control. Endometrial cytology was performed at day 35, immediately before the ozone treatment, and 72 hours later, in both groups. Transrectal ultrasonography and reproductive parameters were also evaluated. It was concluded that the cows treated with ozone showed reduction of polymorphonuclears cells and the prevalence of subclinical endometritis, thus, improving the reproductive performance. Ozone concentration of 50 μg/ml, at a flow of 1 L/min, for 15 minutes has been indicated.

Zobel et al. (2014) distributed 400 cows into two groups, treated with: 20 mL of marketed intrauterine ozonated foam, within 6 hours after calving and 24 hours later; and non-treated animals. The cows were artificially inseminated daily from day 120 after calving, when oestrus was detected. The group treated with ozone had fewer open days, with fewer inseminations, demonstrating that intrauterine ozone after calving can improve fertility.

Constantin and Bîrţoiu (2016) evaluated the effects of intrauterine application of commercial ozone foam in dairy cows with post-partum (7-10 days) endometritis. The control group was formed by cows without clinical signs of uterine inflammation which received no treatment. After an epidural anesthesia, the cows diagnosed with endometritis were treated with a commercially available ozone foam (10 seconds) intrauterine weekly, during a month. After the treatment, all cows were submitted to a hormonal therapy to perform an artificial insemination. The microbiological analysis showed no significant effect of ozone on uterine infection. Nonetheless, the cows treated with ozone had a significative improvement of the first service conception rate and the average of straws until pregnancy.

### 2.4 Mastitis treatments

Argudo and Soria (2017) divided 54 cows with mild and moderate clinical mastitis into 3 groups, where: the control group received intramuscular antibiotics (Ceftiofur 1.6 mg/Kg); a group treated with gaseous ozone intramammary; and the last group received ozonated saline solution intramammary. All the treatments were performed once a day for three days. After 24 hours of the last treatment, the clinical analysis and the presence or absence of flakes and clots in the milk revealed no difference between the group treated with gaseous ozone and the one treated with antibiotics. The cows treated with ozonated saline solution had a significant lower improvement rate. The gas ozone group received 35 μg/mL of gaseous ozone intramammary; the last group received 50 mL of ozonated saline solution intramammary at a declared concentration of 35 μg/mL.

Torrico et al. (2018) selected 73 dairy cows and a total of 165 quarter affected with clinical or subclinical mastitis. They applied gaseous ozone in each mammary quarter, once a day for three days. There was no control group. A California Mastitis Test (CMT), culture and antibiogram test were performed immediately before the first ozone administration and 24 hours after the last one, revealing that 39% of the mammary quarters showed a reduced in the microbial load, while 23% of them showed no signs of mastitis after the ozone administration. They applied 50 mL of gaseous ozone intramammary at a concentration of 35 μg/mL.

In a study performed by Enginler et al. (2015), 32 dairy cows and a total of 79 infected mammary quarters were divided into five groups and treated with: intramammary gaseous ozone at different concentration; only intramammary antibiotics; maximum ozone concentration + antibiotics. All the treatments were performed once a day for one week, after milking of the animals. The animals treated with intramammary antibiotics eventually received intramuscular antibiotics daily for 5 days, according to the antimicrobial agent present. Immediately before the treatment and one day after the last administration, CMT and somatic cell count (SCC) tests were evaluated. High doses of ozone and ozone in combination with antibiotics are the best treatment for mastitis. Intramammary gaseous ozone concentrations were 30, 60, and 70 μg/mL, respectively.

Bignotti (2015) in doctoral program in veterinary clinical sciences evaluated 80 cows treated with: intramammary antibiotics; 5 mL of platelet concentrate (1 × 10^9^ platelet/mL); 5 mL ozonated oil; a blend of 2.5 mL platelet concentrate with 2.5 mL ozonated oil. The last group showed that the synergistic mechanism of ozone and platelets is the best treatment for mastitis, when compared to the other groups, regarding to milk quality and clinical signs of the disease. The ozonation process was performed employing a bubble time of 15 minutes of a 30 mg/L oxygen/ozone gaseous mixture every 100mL of oil. No further information was reported for the ozonated derivative characterization.

### 2.5 Wound healing

A case reported by Garcia et al. (2010a) showed the efficacy of ozone in treating a 15 years old horse with a lesion on the metatarsus, suspicion of cutaneous habronemosis. For that 250 mL of ozonated water and 100 mL of ozonated oil, were immediately applied in its wound, twice a day. A transrectal insufflation in the same conditions above were performed twice a week, initially for 5 minutes, reaching 10 minutes in the last applications. The protocol was performed for 2 months. After that period, it was possible observe an improvement in the skin healing. Ozone derivatives have been both obtained by an ozone generator producing 0.0014g/O_3_/hour in a stream of 1L/min.

Cases reported by Kosachenco et al. (2018) included 4 dogs with big and infected wounds due to polytrauma, treated firstly with antibiotics, analgesics, anti-inflammatories and multivitamin supplement, besides debridement and removal of devitalized tissues and myiasis. For six weeks, all the dogs were submitted to an intrarectal insufflation of gaseous ozone at a concentration 18 μg/mL and volume 2mL/kg, once a week. In two dogs, it was also performed the minor autohemotherapy, where 2 mL of their own blood was mixed with gaseous ozone at a concentration of 25 μg/mL during the first two administrations, increasing to 30 μg/mL the last four ones and applied intramuscularly. For the local administration, two dogs were submitted to the “bagging” ozone at a concentration of 40 μg/mL for 20 minutes every three days, reducing to 20 μg/mL once a week according to their improvement; while the other two dogs received intra and perilesional injections of gaseous ozone at a concentration of 40 μg/mL for the first application and 10 μg/mL for the next ones, twice a week. All the dogs were also treated with ozonated sunflower oil twice a day. It was possible to observe the antimicrobial effect of ozone and a rapid and good granulation tissue, followed by re-epithelization of the wounds.

Repciuc et al. (2020) reported a case of a 12-years-old cat FIV-positive (immunodeficiency virus). The animal also presented a purulent arthritis and, after a failed treatment, had that limb amputated, followed by and rejection of the surgical material, skin necrosis and wound dehiscence. The ozone therapy started 13 days after the amputation, every 48 hours for 38 days. After the first session, the borders of the wound started to contract and exudates were significantly reduced, and after 40 days of the beginning of the treatment, the wound surface was completely healed. Ozone perilesional and intralesional infiltrations were performed at a concentration of 15 μg/ml. The volume administrated was 1.0–1.5 ml of gas subcutaneously, perilesional infiltrated at an average 2–3 cm distance between points and 2 cm distance from the border of the wound.

Cezario (2018) in her thesis dissertation reported a case of a recurrent skin wound in a 6-months-old cat. After the conventional antibiotic treatment and a surgical intervention for debridement and cleaning, the animal presented lameness. Despite decreased bone density and contours irregular of the femur, the bone biopsy concluded there was no alterations. After five daily intralesional ozone sections, the skin wounded and it was possible to observe bone improvement in the radiography. No more information about what application modality or ozone concentration has been given.

### 2.6 Foot rot

A study performed by Szponder et al. (2017) selected 15 sheep, which 10 were suspected of foot rot and 5 were healthy. The sheep from foot root group were submitted to a cleaning and removal of necrotic tissue, application of dressing, which was infused with 500 mL ozonated saline solution. After that, the bandages were removed. This protocol was performed once a week for three weeks. In case of non-healing, animals were treated with activated platelet rich plasma (PRP). 60% of the animals was completely healed after the ozone administration, while the other 40% demonstrated a full recovery after the PRP treatment. No hematological parameters had changed, but it was possible to observe a significant increase in antiradical activity in the groups treated with ozone. They concluded ozone is safe and effective in foot rot treatment, especially when combined with application of PRP. The authors state that “the therapeutic solution was prepared using a medical generator for ozone therapy which supplied 500 mL of 0.9% NaCl with a concentration of ozone of 70 mg/mL”.

### 2.7 Laminitis

Coelho et al. (2015) reported a case of a horse diagnosed with Obel grade IV chronic laminitis on the right foot. The protocol exclusively included corrective trimming and ozone therapy: 10 mL of peritendinous ozone (19 μg/mL); 10 mL of intramuscular ozone at various points of the anterior limb; intrarectal insufflation for 5 minutes (5 – 39 μg/mL) twice a week, for 10 weeks. The animal was also submitted to an osmotic footbath and drainage of an abscess. After the treatment, the animal improved from grade IV to grade II. Six months later, the horse showed a better body condition and ambulation, despites being still grade II, with no signs of infection and a normal relationship between dorsal hoof wall and the distal phalanx.

### 2.8 Equine joints

Vendruscolo et al. (2018) selected 14 clinical healthy horses, totalizing 24 tibiotarsal healthy joints, which were divided into three groups, randomly treated with: 15 mL of O_2_; 15 mL of gaseous ozone at a concentration of 20 μg/mL; and 40 μg/mL, respectively. Each joint was treated 10 times, with an interval of 15 days between the applications. Besides no significant differences of biomarkers of inflammation and cartilage catabolism, which proves the safety of the application, it was concluded that consecutive treatments can cause mild lameness and transient changes in ultrasonography.

Silva et at. (2020) used infrared thermography to diagnose a non-infectious inflammatory process of a horse, posteriorly treated with five applications of 120 mL of intramuscular ozone in the scapular area and interval of three days. Ozone was able to reduce the surface temperature, since it reduced also the inflammatory process. In the original article, the authors literally state that “an ozonizer with an oxygen concentrator at 92% (10 L min^−1^), maximum ozone generation of 15 g of O3 at 8 min L-1 of O_2_ with a static mixer injection system/diffuser, bypass and one-inch venturi injector, was used”.

### 2.9 Ophthalmology

Spadea et al. (2018) reported three cases where they used a marketed eye drops containing ozonated oil in liposomes plus hypromellose to treat spontaneous ocular pathologies. We believe it is important to immediately point out that the Authors’ assumption “ozonated oils have the same properties of gaseous ozone” is not quite correct.

The first case is a 26-years-old horse with exophthalmos due to retrobulbar neoformation (probably neoplasm/osteosarcoma) and recurrent conjunctivitis, treated with antibiotics, but without improvement. One day after beginning the therapy with ozone-based eye-drops three times a day, blepharospasms disappeared; after 3 days, the animal had no sign of blepharitis and conjunctivitis and the eye was completely normal after one week.

The second case is a 6-months-old cat affected by chronic conjunctivitis present from birth, positive for *Staphylococcus spp*. and *Enterococcus spp*. After dropping ozone-based collyrium in both eyes, twice a day, it was possible to observed a conjunctival bacterial count normal in both eyes, which had no more symptoms after ten days.

The last case if a three-years-old bulldog affected with chronic keratitis treated with ozone-based eye-drops twice a day. Besides entropion and mucous discharge were still present, after 10 days, keratitis had almost disappeared and corneal edema was resolved.

Zamora et al. (2018) divided 40 rabbits affected with keratoconjunctivitis infected by *S. aureus* and *E. coli* into two groups: the first one received a conventional treatment, with Queratofural, a veterinarian collyrium; while the second one was treated with ozonated oil-based collyrium. Both treatments were performed once a day for seven days. After the treatment, the microbiological analysis was negative in both groups, proving the efficacy and safety of the protocol. No information is provided regarding the characterization of the ozonated sunflower oil.

### 2.10 Oncology

Hernández Avilés et al. (2016) reported four cases of dogs with different oncological process (lymphosarcoma, chondrosarcoma, adenocarcinoma and osteosarcoma) treated with ozone therapy. Three dogs were also submitted to chemotherapy and one to surgery. However, when the animals received both ozone and chemotherapy, it is not possible to deduce a clear understanding of the positive effect of the application of ozone.

In the first case, besides the lymphosarcoma, the dogs which was also Leishmaniosis-positive, was treated with 3mL/Kg intrarectal insufflation (15-35 μg/mL) and minor autohemotherapy (10-30 μg/mL) for five months, while it received also chemotherapy. After 30 months, the animal has an excellent quality of life and is still under remission.

In the second patient, affected by a chondrosarcoma, besides the chemotherapy, they performed major autohemotherapy, applying intravenously 1 mL of blood/Kg mixed with an equal volume at ozone at a concentration of 20 μg/mL; 40 mL of gaseous ozone at a concentration of 15 μg/mL intra- and periarticular in the hip joint. The dog had been fine for 19 months, when started limping again. The therapy was applied again, with improvement, but after two months, the animal died, apparently from natural causes.

The adenocarcinoma of the thyroid gland case was treated with 7 consecutive sessions of gaseous ozone infiltration intratumorally at a concentration of 30 μg/mL and 3mL/Kg intrarectal insufflations at a concentration of 20 μg/mL. The tumor decreased 20% of its original size and the animal had an excellent quality of life until its death, 7 months after the diagnosis.

The last case, besides the chemotherapy and a surgery, intralesional ozone was infiltrated at a concentration 8-15 μg/mL, along with 3 mL/kg of rectal insufflation at concentration of 20-30 μg/mL. After 4 years of the diagnosis and remission, the animal is still submitted to 4 cycles per year of intrarectal ozone therapy and presents an excellent quality of life.

Gayon-Amaro and Flores Colin (2019) treated 5 dogs affected by oncological process (mammary adenocarcinoma, vaginal adenocarcinoma, basal cells tumor in the scrotum, osteosarcoma and melanoma in the lower eyelid) with local infiltration, topical instillation of ozonized oil, major and minor autohemotherapy and rectal insufflations. No other information available. It was observed a general improvement of quality of life of all patients, being 2 in complete remission, one with decreasing size of tumor, another with apparently inactivation and the last case with no clinical signs.

### 2.11 Infectious diseases

Gonçalves et al. (2020) related a case of a dog tested positive for leishmaniosis. Besides domperidone 1 mg/kg (twice a day – 30 days), alopurinol 15 mg/kg (twice a day – 30 days) and miltefosin 2% (0,1 mL/kg daily for 28 days), the dog was also submitted to an ozone protocol ending after 12 sessions. After the 4^th^ application it was possible to see improvement at the skin wounds. Besides the clinical improvement, it was also possible to notice reduced on the side effects of the drugs, improvement of immune response and healing of the skin. Ozone application started with 60 mL intrarectal insufflation of ozone (20 μg/mL), minor autohemotherapy (20 μg/mL) and perilesional ozone infiltration (14 μg/mL). The latest applications have covered only autohemotherapy and intrarectal administration.

Cabral et al. (2020) infected 72 mice with *Leishmania amazonensis* and divided them into 6 groups, treated with: meglumine antimoniate intraperitoneally once a day for 30 days; ozone topical treatment of the infected paw, submerged for 5 minutes once a day for 30 minutes; meglumine antimoniate + topical ozone saline; gaseous ozone administrated intraperitoneally 3 times a week, for 30 days. There was also one group non-infected and non-treated; and one infected and non-treated. All the treatments had significant reduction of the lesions, especially when treated with meglumine + topical ozone. It was also possible to observe better wound healing and immunomodulatory activity in animals treated with ozone. Besides, promastigotes of the parasites were incubated *in vitro* and treated with different concentrations of ozonated saline solution (from 1 up to 15 μg/mL). 300 μg/mL of meglumine antimoniate were used as positive control. After 72 hours, it was possible to observe a significant reduce in the number of parasites in all concentrations, which 15 μg/mL was similar to the positive control, demonstrating leishmanicidal capacity of ozone *in vitro*. For topical treatment, 20 μg/mL was bubbled into the saline solution for 5 minutes, while for systemic one 30 μg/mL of gaseous ozone have been intraperitoneally administrated.

A case reported by Garcia et al. (2010b) treated a two-years old dog tested positive for *Ehrlichia sp.* with major autohemotherapy, by mixing 80 mL of the patient's blood (8% of its corporal weight) with the same volume of gaseous ozone. The ozonated blood was reinjected into the jugular or radial vein, and the process was repeated two-three times a week. Through blood samples collected before and after the treatment and the clinical evaluation, it was possible to notice an effective reversal of ehrlichiosis. The Authors literally state that “medical ozone was produced by a generator with a production capacity of 0.00023 g/min, powered by a cylinder of oxygen with 99.5% purity at a pressure of about 250 kgf/cm2 with a flow of 3 L/min”.

### 2.12 Thrombocytopenia due to hemoparasitosis

Gonçalves et al. (2020) reported a case of a 10-month-old dog with persistent thrombocytopenia, probably due to ehrlichiosis. After the unsuccessful conventional treatment with antibiotics and blood transfusion, ozone therapy was performed. For that, 250 mL of 0.9% NaCl was ozonated for 4 minutes and injected intravenously, and 20 mL of gaseous ozone at a concentration of 13 μg/mL was applied intrarectally. The ozone therapy was performed only once and after 15 days, the animal presented a normal blood count and normalization of the clinical parameters.

## 3 Discussion

In the veterinary field, we still have lot of scientific publications with missing or incomplete data, poor case reports and papers without a control group or often-unrepresentative sample (Briel et al., 2013). The Vet applications of ozone and its derivatives are numerous and very interesting. However, in consideration of the multiplicity of methods of ozone administration and the scarcity of veterinary clinical trials (Oyama et al., 2017), the compliance with the highest quality standards in publications about ozone is even more important.

**Fig. 1:**
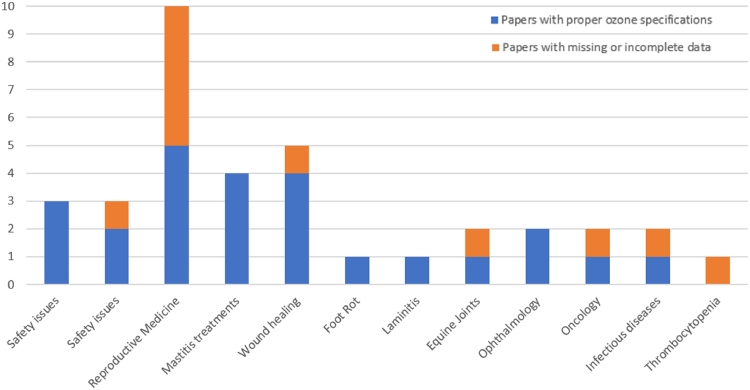
Grouping of the original papers according to the ozone specifications with respect to the topics.

In order to offer an immediate overview, in Fig. 1 the overall situation of the articles reviewed up to now and grouped by similarity of ozone treatment modalities is schematically represented. As it is possible to observe, there is a considerable variability on the descriptive appropriateness of the methods of administration of ozone or its derivatives. However, most of the papers adequately mention the specifications relating to the use of ozone.

It is worth mentioning that some reports, even though they are innovative and very important, they are published in journals with low international spread and often in a language other than English, which hinders the delivery of this information globally.

**Fig. 2:**
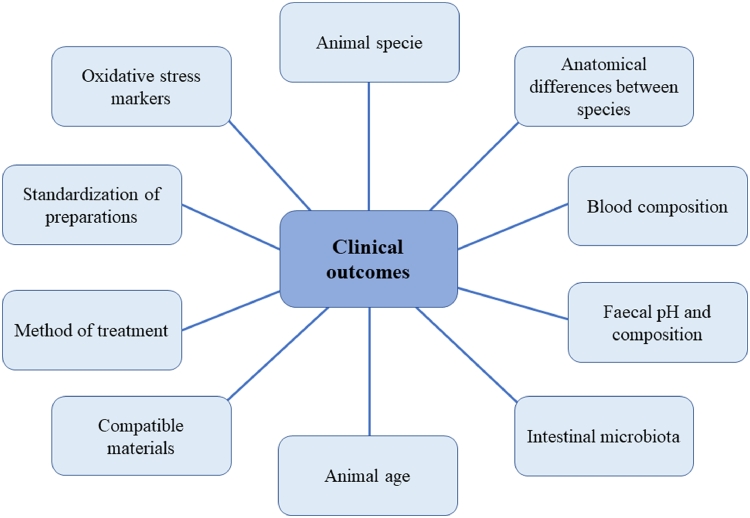
Multiplicity of factors impacting on ozone therapy clinical outcomes.

On the other hand, the features to be taken into consideration in the case of the use of oxygen-ozone gas mixtures for therapeutic purposes are many. A summary of the factors that can interfere with ozone administration are highlighted in Fig. 2. All these aspects must be taken into consideration for an overall evaluation of the clinical outcomes deriving from the therapeutic application of ozone and its derivatives.

A correct interpretation of the following aspects opens very important avenues of research in terms of the development of ozone therapy, enabling improvements in the design of clinical trials and more precision in interventions in the treatment of the various veterinary diseases.

### 3.1 Blood composition

For a suitable systemic ozone evaluation, differences between animal and human blood composition have to be taken into consideration. It is also important to notice that the human hematological and biochemical range are different when compared with the animals, which also diverges according to the gender, age, species, breed, and the altitude that those animals are (Miglio et al., 2020; Mortola and Wilfong, 2017; Scholkmann et al., 2019; Wintrobe and Shumacker, 1936). For example, while in dogs, the hematocrit references value are between 37-55% in dogs, 24-45% in cats, 32-48% in horses and 41-54% in humans; the blood cell distribution in M/mm³ is 5-8,5 in dogs, 5-10 in cats, 6-12,9 in horses and 4,3-6 in humans (Klaassen, 1999; Soares et al., 2012). There are also differences between venous and arterial blood, which reinforces the importance of the method of blood collection for the application of ozone therapy (Lee et al., 2020). All these aspects are of fundamental significance, especially concerning the use of autohemotherapy, both in terms of M-AHT (Chica, 2020; Garcia et al., 2010b; Kosachenco et al., 2018) and m-AHT (J. O. S. Gonçalves et al., 2020; Kosachenco et al., 2018).

### 3.2 Systemic treatment methods

It should be noted immediately that both M-AHT and m-AHT methods are the only ones to guarantee standardization in terms of operative procedures for sampling, quantity of blood, quantity of gaseous ozone at a certain concentration, blood contact time, administration. In fact, ozone solubility and disappearance profile are mainly related to the presence of solutes, like 0.9% sodium chloride (Bocci et al., 2012; Cabral et al., 2020). This aspect does not guarantee the concentration of solubilized ozone which is infused systemically (Szponder et al., 2017). In fact, the method of insufflation, the ionic strength, the composition of the solutions and the time elapsed between preparation and administration significantly modify the ozone present. Moreover, an even more important difference is related to the different ways in which ozone reacts with biological fluids. In fact, the blood administration of solubilized ozone in infusional liquids leads to the intravascular reaction drop by drop, with consequent *in vivo* formation of the effector molecules in dynamic mode along the circulatory stream. Similar considerations concern the subcutaneous (Roman, 2015) and intramammary (Argudo and Soria, 2017) administration of these solutions or of ozonated distilled sterile solution at intrauterine level (Escandón et al., 2020; Maldonado et al., 2017). However, in these cases local action prevails and, therefore, the systemic implications are less important or unwanted. Furthermore, some practitioners produce their own ozonated solution or autohemotherapy, in the absence of information regarding the compatibility of the material used (Argudo and Soria, 2017; Cabral et al., 2020; Chica, 2020; Escandón et al., 2020; Garcia et al., 2010a, 2010b; Gayon-Amaro and Flores-Colin, 2019; Gonçalves et al., 2020; Hernández Avilés et al., 2016; Maldonado et al., 2017; Roman, 2015; Szponder et al., 2017). On the contrary, it is well-known that blood ozonation into PVC bags can stimulated the discharge of plasticizers (Ciborowski et al., 2012), which could be harmful for the patients.

Morever, gaseous ozone is also used by rectal insufflation to achieve a systemic effect (Jaramillo et al., 2020). While the human colon is sacculated, canine colon does not have sacculation. The total gastrointestinal transit velocity in humans is 20-30 hours, while in dogs it is 6-8 hours. The equine cecum represents up to 15% of the gastrointestinal capacity, while in dogs represents less than 2%. Feces pH is 6,2 in dogs; 7,0 in cats; and 7,5 in horses and cows. Colon pH is 5,5-7 in humans; 6,5 in dogs; 6,2 in cats; and 7,4 in horses and cows. In a comparative study between different species, Kararli concludes that “no single animal can mimic the gastrointestinal characteristics of humans” (Kararli, 1995). This data is relevant especially when intrarectal administrations are performed, because different anatomical, physiological and biochemical gastrointestinal differences might lead to a different ozonolysis products and absorption of effector molecules.

### 3.3 Topical treatment methods

Topical or loco-regional action can be considered for the administration of gaseous ozone at the level of the joints (Argudo and Soria, 2017; Enginler et al., 2015; Repciuc et al., 2020; Torrico et al., 2018; Vendruscolo et al., 2018) or intramuscular (Silva et al., 2020). In these cases, the modality by which it has been carried out is of paramount importance, mainly in terms of safety of the treatment (Bonetti and Travagli, 2020).

The evaluation of the results obtained in the case of combinations of treatments it becomes even more difficult to establish standardization criteria (Cabral et al., 2020; Coelho et al., 2015; Garcia et al., 2010a; Gayon-Amaro and Flores-Colin, 2019; Gonçalves et al., 2020; Hernández Avilés et al., 2016; Kosachenco et al., 2018).

Eventually, it is important to remember the use of ozonated derivatives starting from vegetal matrices, both in the form of laboratory-obtained (Madan et al., 2010; Zamora et al., 2018), and commercially available products (Constantin and Bîrţoiu, 2016; Daud et al., 2011; Djuricic et al., 2015, 2012; Đuričić et al., 2016, 2014; Polat et al., 2015; Rodríguez et al., 2019; Spadea et al., 2018; Zobel et al., 2012, 2014). In fact, there are many different forms of commercial ozone preparations (eg, foam, pearls, boluses, injections, cream, eye-drops). In these cases, other than the properties of the commercial preparations, the standardization of treatment methods and times influence the results obtained. As regards the explicit indication of the peroxide index, it must be emphasized that there is still no specific method capable of giving reproducible and official results (Bignotti, 2015; Kosachenco et al., 2018).

## 4 Topical features

### 4.1 Skin burns - Pantanal

In 2020, in Pantanal, the Brazilian most flooded biome, until 15^th^ November, 4,3 million hectares (more than 30% of the total area) has been burned. The area of fire outbreaks had increased more than 80% when compared to the last year (Libonati et al., 2020). This whole ecosystem is being destroyed, including the Parque Estadual Encontro das Águas, where the highest concentrations of jaguar on the planet inhabits. Besides countless wildlife who already lost their lives, many animals were rescued with very serious burn (Spring, 2020). Recently, one jaguar has been treated with ozone therapy and laser, which has helped its wound healing and accelerating its recovery, allowing its return to the nature after two months (Martins and Santos, 2020; Santana, 2020).

These results agree well with what has long been demonstrated by Valacchi et al. (2011, 2013) and Travagli et al. (2010) according to which ozone derivatives deplete oxidant levels, increase oxidative markers and induce redox sensitive transcription factors, Heat Shock Protein (HSP) and Matrix Metalloproteinases (MMPs). Besides improving wound healing, reducing pain and edema and provide a better graft retention, ozone can also prevent skin infections, that are very common in burns (Peretyagin and Struchkov, 2013). Decreasing the rehabilitation period is especially important in wild animals, due to economic resources and animal welfare (Sleeman, 2008).

### 4.2 SARS-CoV-2 in animals

The origin of SARS-CoV-2 is probably animal (Andersen et al., 2020), which makes it a zoonotic pathogen. SARS-CoV-2 PCR was also detected in dogs (Patterson et al., 2020; Sit et al., 2020), cats (CDC, 2020; Patterson et al., 2020; Ruiz-Arrondo et al., 2020; Zhang et al., 2020), tigers (McAloose et al., 2020; USDA APHIS, 2020), lions (McAloose et al., 2020) and minks (Oreshkova et al., 2020). Experimental infections were tried in monkeys, cynomolgus macaques, rhesus macaques, ferrets, hamsters, mice, tree shrew, pigs, poultry, dogs, cats and bats (Abdel-Moneim and Abdelwhab, 2020).

Some animals, like pigs and poultry do not seem to be vulnerable to the virus (Abdel-Moneim and Abdelwhab, 2020; Johansen et al., 2020), while dogs, for example, demonstrated low susceptibility. On the other hand, felines seem to be more susceptible, demonstrating even some symptoms (Abdel-Moneim and Abdelwhab, 2020; CDC, 2020; McAloose et al., 2020; Newman et al., 2020).

Despite its use in SARS-CoV-2-infected environment, clinical trials using ozone therapy for treating humans infected with SARS-CoV-2, demonstrated that patients treated with ozonated autohemotherapy or rectal ozone had decreasing in the level of inflammation biomarkers, improved oxygen saturation and radiological signs and, finally, clinical improvement associated with a significantly shorter hospital recovery time (Fernández-Cuadros et al., 2020; Hernandez et al., 2020; Hernández et al., 2020). Therefore, this is a therapy that could be useful also in animals, especially those who demonstrated respiratory symptoms. It is worthwhile reinforcing that no evidences that animals can transmit the virus to human been was declared (Abdel-Moneim and Abdelwhab, 2020; Almendros and Gascoigne, 2020; Wang et al., 2020).

## 5 Conclusions

The considerable variability in the adopted practices limits standardization and it can justify the different and opposing findings found in the literature. The use of ozone therapy in animals must be performed by veterinarians with a specific preparation from veterinary schools delivered at University level. The degree in one of the courses above-mentioned is a fundamental and essential element for practicing this promising animal healthcare service. Design a network for including private veterinary practices in randomized controlled trials is also a conceivable upgrade. All with the aim of taking nothing away to the cited original research papers, but of improving acceptance of ozone as a generator of effector molecules also useful in the multiple fields of Veterinary and animal science.

## Research funding

This study was financed in part by the 10.13039/501100002322Coordenação de Aperfeiçoamento de Pessoal de Nível Superior – Brasil (CAPES) – Finance Code 001; and 10.13039/501100001807Fundação de Amparo à Pesquisa do Estado de São Paulo (FAPESP) – grant number 2018/24552-6.

## Ethical statement

Not applicable

## Declaration of Competing Interest

The authors declare that they have no known competing financial interests or personal relationships that could have appeared to influence the work reported in this paper.
